# Evaluation of cycloserine dose regimens in an Indian cohort with multidrug-resistant tuberculosis: a population pharmacokinetic analysis

**DOI:** 10.1128/aac.00101-25

**Published:** 2025-09-02

**Authors:** Juan Eduardo Resendiz-Galvan, Prerna R. Arora, Rohan V. Lokhande, Zarir F. Udwadia, Camilla Rodrigues, Amita Gupta, Jeffrey A. Tornheim, Paolo Denti, Tester F. Ashavaid, for the The MDR-TB MUKT and Indo-South Africa Study Teams, Lancelot Pinto

**Affiliations:** 1 Division of Clinical Pharmacology, Department of Medicine, University of Cape Town 71984, Cape Town, South Africa; 2 Research Laboratories, P. D. Hinduja National Hospital and Medical Research Centre 29537, Mumbai, India; 3 Center for Infectious Diseases in India, Division of Infectious Diseases, Johns Hopkins University School of Medicine 1500, Baltimore, Maryland, USA; 4 Center for Tuberculosis Research, Division of Infectious Diseases, Johns Hopkins University School of Medicine 1500, Baltimore, Maryland, USA; 5 Bloomberg School of Public Health, Johns Hopkins University School of Medicine 1500, Baltimore, Maryland, USA; City St George's, University of London, London, United Kingdom

**Keywords:** MDR-TB, cycloserine, pharmacokinetics, NONMEM, pharmacometrics

## Abstract

Cycloserine is recommended for inclusion in regimens for multidrug-resistant tuberculosis (MDR-TB). Its efficacy is time dependent and relies on the concentration remaining above the minimum inhibitory concentration (MIC); however, there is a concentration-dependent risk of neurotoxicity. Limited pharmacokinetic (PK) data are available in individuals of Indian origin, despite the high burden of MDR-TB in India. We enrolled adults and adolescents receiving cycloserine for MDR-TB at a tertiary hospital in Mumbai, India, in a prospective cohort. Total daily doses ranged from 500 to 750 mg, and participants underwent serial PK sampling on multiple visits starting 1 month after treatment initiation. PK data were analyzed using non-linear mixed-effect modeling. A total of 180 participants (117 females) were enrolled, with a median age of 27 years (interquartile range [IQR] 21–35), weight of 56.0 kg (IQR 46.0–65.9), and fat-free mass of 38.6 kg (IQR 32.3–47.1). Cycloserine PK was best described by a one-compartment model with first-order elimination and transit compartment absorption. Allometric scaling by fat-free mass provided the best adjusment for body size. Serum creatinine improved the model fit and allowed separate estimation of renal and non-renal clearances, whose typical values were 0.589 and 0.901 L/h, respectively. Simulations showed median exposure of 308 mg·h/L after 250 mg twice daily (BID), which is lower than reported in literature. Monte Carlo simulations suggested that doses of 500 or 750 mg BID are required to reach efficacy targets of ≥30% and ≥64% time within dose interval above MIC. The reasons behind the low exposure identified in this Indian population require further investigation.

## INTRODUCTION

India faces a difficult challenge combating the emerging scourge of multidrug-resistant tuberculosis (MDR-TB), with the highest global burden according to the World Health Organization (WHO) (1). To counter this epidemic, Indian national guidelines include cycloserine as a core drug in longer MDR-TB regimens (2), which are recommended in the face of complex circulating resistance to drugs in newer and shorter MDR-TB treatments (3). The inclusion of cycloserine in MDR-TB regimens is associated with improved treatment success and reduced mortality (4), and population studies have found lower rates of cycloserine-associated toxicity than expected in India (5), suggesting an opportunity to optimize efficacy using local population pharmacokinetic (PK) data.

Cycloserine can be dosed as either cycloserine itself or its analog, terizidone. The recommended dosage is 500 mg daily for individuals with a body weight of ≤45 kg, 750 mg daily for those >45 kg (6), or up to 1,000 mg daily for those ≥70 kg (2). Split dosing is advised to reduce toxicity. Absorption may be reduced in the presence of a fatty meal (6), but cycloserine otherwise exhibits rapid absorption upon oral administration, reaching peak plasma concentrations in approximately 0.84 h, and has a terminal half-life of about 13 h (7). Cycloserine is considered not to bind to serum proteins (8) and undergoes minimal hepatic metabolism, with 60%–70% excreted unchanged by the kidneys (9).

Hollow-fiber data suggest that the most reliable efficacy indicator for cycloserine is the percentage of time during which plasma concentration exceeds the minimum inhibitory concentration (%*T*_>MIC_). Cycloserine is bactericidal when *T*_>MIC_ exceeds 30%, while *T*_>MIC_ of ≥64% achieves 80% of maximal kill (10). Dosing strategies must balance the desire to maximize efficacy against dose-dependent neuropsychiatric toxicity and peripheral neuropathy. Although there is no consistently reported level at which toxicity develops, the likelihood of peripheral neuropathy has been linked with high cycloserine exposure (11).

Few studies have investigated the PK of cycloserine either when dosed as cycloserine (12, 13) or as its dimer, terizidone (14, 15), with limited available data from India. To evaluate the probability of target attainment (PTA) across the dosing range for cycloserine, this study analyzed cycloserine serum concentration data collected from a cohort study of adults and adolescents treated for MDR-TB at a tertiary care center in Mumbai, India.

## MATERIALS AND METHODS

### Study population

Data were collected from a prospective observational study of adolescents and adults with MDR-TB at a tertiary hospital in Mumbai, India (5, 16–18). From October 2017 to February 2022, MDR-TB treatment-naïve individuals aged ≥15 years were enrolled and followed for clinical outcomes and treatment-associated side effects during 24 months of multidrug treatment following both Indian and WHO guidelines and accounting for each individual’s drug susceptibility test results (2). The inclusion of cycloserine and the dose prescribed was left to the discretion of treating clinicians. All participants in the study received cycloserine in the form of Lupin capsules, available in 250 and 500 mg strengths. The choice of cycloserine treatment was largely dependent upon the extent of drug resistance, allowing for at least four effective drugs to be included in a multidrug treatment regimen, with the dose of cycloserine initiated at 250 mg once daily (QD) and increased as tolerated to a target total daily dose of 500 or 750 mg following weight-based dosing bands outlined in the Indian national guidelines (2). Treatment was provided either as equally divided twice-daily (BID) or thrice-daily (TID) regimens or as a 750 mg total daily treatment, administered as 250 mg in the morning and 500 mg in the evening. Drug-level testing was performed for each participant at least 1 week following initiation of target dose.

Throughout the study, participants were routinely monitored to record clinical characteristics, laboratory and imaging results, side effects, and treatment outcomes.

### PK and minimum inhibitory concentration data collection

PK sampling visits were performed at 1, 2, 6, and 12 months after treatment initiation, but due to the impact of the coronavirus disease 2019 pandemic, PK visits were allowed to be rescheduled through the 15th month of MDR-TB treatment. At each PK visit, blood samples were collected from study participants just before and 2 h after the observed cycloserine dose. The first participants enrolled were asked for additional consent to collect intensive blood samples at one of their PK visits (either 1 or 2 months into treatment), and blood was drawn at pre-dose, 1, 2, 4, 6, and 8 h after dose. Blood samples were collected in EDTA whole blood and centrifuged at 3,000 rpm for 10 min. Plasma was then collected, aliquoted, and stored at −80°C until analysis at the Hinduja Hospital laboratory. Quantification employed an in-house developed and validated high-performance liquid chromatography coupled with a tandem mass spectrometry method with linearity in a range of concentrations from 0.782 to 50 mg/L. The interday accuracy and precision ranged from 98.3% to 107.5% and from 1.8% to 10.2%, respectively.

Minimum inhibitory concentrations (MICs) were determined by culture of pre-treatment sputum samples. Diagnostic specimens were submitted for liquid culture using the Mycobacteria Growth Indicator Tube (MGIT) system. Positive isolates were then cultured again on Löwenstein–Jensen media until late-log phase growth and saline agitation for inoculation on custom Sensititre plates using a Scientific AIM auto-inoculator (Thermo Fisher). Isolates were tested at cycloserine concentrations of 2, 4, 8, 16, 32, and 64 mg/L, which were quality-controlled using two drug-free control wells on each plate and parallel testing of the H37Rv laboratory strain on an identical plate with each batch to confirm expected results.

Plates were read using a mirror box on days 10, 14, 21, and 28 following inoculations by two independent readers to ensure concordance, with the final value selected as the first date with adequate growth in both drug-free control wells. When the two readers reported discordant values, a third independent reader adjudicated the result.

### Model development and PK analysis

Cycloserine plasma concentrations were analyzed with non-linear mixed-effect modeling using the software NONMEM v.7.5 (ICON Development Solutions, Hanover, MD, USA) (19) and the algorithm first-order conditional estimation with interaction Graphical diagnostics, management, and organization of the model were handled using Perl-speaks-NONMEM, Xpose4 (20) embedded in R, and Pirana (Certara, Princeton, NJ, USA) (21), respectively.

Initially, the modeling process focused on the intensively sampled data. Once the model accurately described these intensive data, the sparsely sampled data were incorporated for reassessment of PK parameters and to conduct additional covariate testing, utilizing the complete data set. Various structural models were investigated, including one- and two-compartment dispositions, together with first-order absorption with lag time or transit compartments (22). Between-subject variability and between-occasion variability (23) were assumed to follow a log-normal distribution. Between-subject variability was evaluated on all disposition parameters, and between-occasion variability was added for all absorption parameters, including bioavailability. For between-occasion variability estimation, non-observed doses before the PK visits and pre-dose concentrations were considered as an independent occasion, separate from the observed doses during the visits and the subsequent concentrations. Residual unexplained variability was modeled testing both additive and proportional components. Concentrations below the limit of detection (BLD) were censored by the laboratory and incorporated into the model using an adaptation of the M6 method (24). In summary, BLD concentrations were imputed as limit of detection (LOD)/2 (0.195 mg/L), and the additive component of RUV was inflated by LOD/2 to account for the additional uncertainty introduced by imputation. If a series of consecutive BLD concentrations was found, only one value was included in the model fit (the last if in the absorption phase and the first one if in the elimination phase) but retained for simulation-based diagnostics.

Allometric scaling was included from the initial phases of the modeling process to describe the effect of body size on disposition parameters and was tested using total body weight or fat-free mass (FFM) (estimated based on sex, height, and body weight) (25) as body size descriptor. The effect of physiologically plausible covariates on PK parameters was evaluated using a stepwise approach and inspecting improvement in model diagnostics, including goodness-of-fit plots and visual predictive checks. Forward inclusion of covariates required a NONMEM objective function value reduction (ΔOFV) of ≥3.84 units for inclusion of 1 degree of freedom (df) (*P* < 0.05), followed by backward elimination with a ΔOFV of ≥6.63 units for retention in the model of 1 degree of freedom (*P* < 0.01). Categorical covariates considered for the model included sex, self-reported smoking status, medication taken with food during PK visit, and additional drugs prescribed. Continuous covariates tested in model assessments were age, serum creatinine, Cockcroft–Gault creatinine clearance estimates (26), and days on cycloserine treatment.

To minimize the overlapping effects of sex and body weight on the calculations of both renal elimination and allometry by FFM, we adapted the approach suggested by Holford et al. (27) to separate renal and non-renal contributions to cycloserine clearance (CL). We first employed the Cockcroft–Gault formula using each individual’s age and serum creatinine but removing the effect of weight and sex by using the median value of weight in the cohort (56 kg) and using the formula for males for every patient, as shown below:


$$Cockcroft\text{-}Gault\;{CL}_{cr,56M}=\frac{(140-Age\;in\;years)\cdot 56\;kg}{72\cdot SCr\;(mg/dL)}$$

where CL_cr,56M_ represents the Cockroft–Gault creatinine clearance estimates for a 56 kg weight male with individual values of age and serum creatinine.

Then renal function (RF) was estimated by normalizing CL_cr,56M_ by its median in the population$\left(\bar{{CL}_{cr,56M}}\right)$


$$RF=\;\frac{{CL}_{cr,56M}\;}{\bar{{CL}_{cr,56M}}}.$$

Therefore, an individual with a ${CL}_{cr,56M}$ equal to the median in the population would have an RF of 1.

Finally, the CL was divided into its renal (CLr) and non-renal (CLnr) clearances by multiplying the renal component by RF. Finally, the effect of body size Fsize (as per allometry) is applied to the overall CL (CLr + CLnr).


CL=(CLnr+RF⋅CLr)⋅Fsize.

The uncertainty of parameter estimates for the final model was determined using the sampling importance resampling method (28).

### Simulations

The final model was used to perform Monte Carlo simulations in a virtual population generated from repetitions of the original data set for a total of 1,800 individuals. This data set was used to evaluate the probability of attaining a *T*_>MIC_ of ≥30% and ≥64% related to bactericidal activity and 80% of maximum kill, respectively (10). PTA was evaluated under the six regimens received by study participants: 250 or 500 mg QD, 250 mg BID or TID, 250/500 mg a.m./p.m., and 500 mg BID. Two additional regimens were simulated: WHO-recommended weight-based dosing of 250 mg BID for weights ≤45 kg and 250/500 mg a.m./p.m. for body weights >45 kg, and a flat dose regimen of 750 mg BID. The %*T*_>MIC_ was evaluated separately for each dose and frequency regimen under the assumption that cycloserine was not protein bound in plasma (i.e., unbound fraction = 100%). The different dosing scenarios were simulated for the median, first, and third quartile values of RF observed in our data.

### Exposure and toxicity

Observed and simulated exposures were compared to values reported in the literature. In order to evaluate the extent to which the literature-derived toxicity threshold for area under the concentration curve (AUC_0–24_) of ~700 mg·h/L (11) was clinically significant in this cohort, participants were dichotomized as below or above the threshold and with or without cycloserine-associated toxicity, including self-reported depression, neuropathy, or psychosis, as well as abnormal 5 g monofilament or vibration sense testing, and elevated Patient Health Questionnaire-9 (PHQ-9) scores (29). Differences were evaluated by chi-squared or *t*-tests for categorical variables and *t*-tests for PHQ-9 scores. Additionally, we evaluated the estimated AUCs (both as raw and log2 normalized) as continuous variables and performed univariate logistic regression (glm function in R) against simultaneous report of the same toxicities. Finally, we used the pROC package in R (30) to calculate Youden’s best threshold to use AUC to discriminate toxicities.

## RESULTS

### Baseline characteristics

**Fig 1:**
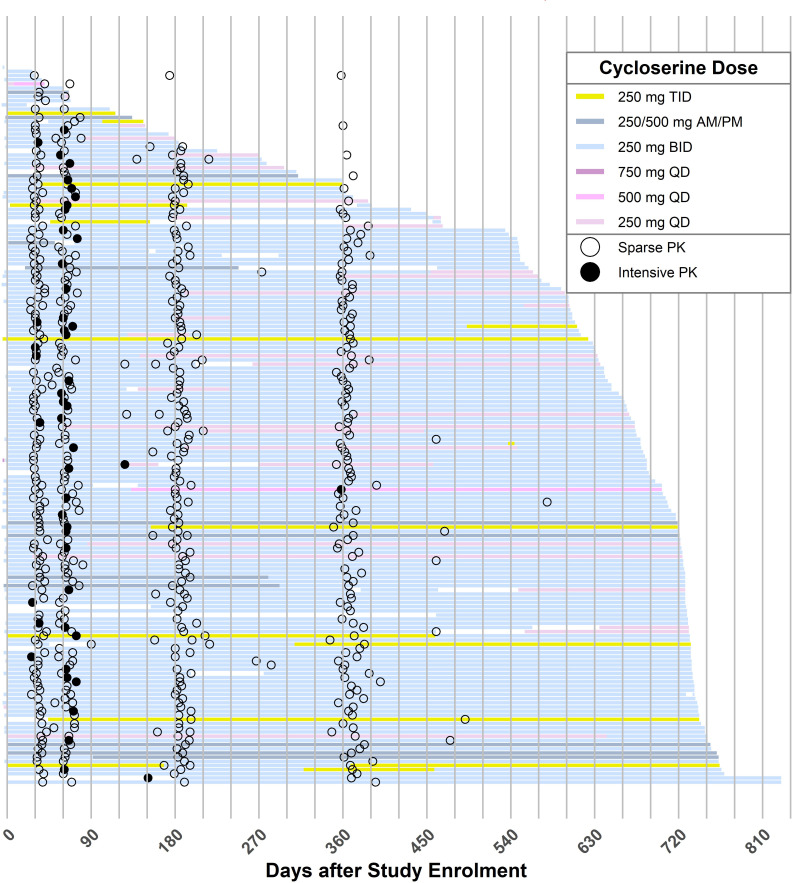
Duration of cycloserine treatment prior to drug-level testing. Each horizontal line represents a single participant’s cycloserine treatment duration, with the color coding denoting the total daily cycloserine dose prescribed over that period of time. Filled circles indicate intensive sampling visits, and open circles indicate sparse sampling visits.

A total of 180 participants, 117 of whom were female, provided cycloserine PK data, with a median age of 27 years (interquartile range [IQR] 21–35), weight of 56.0 kg (IQR 46.0–65.9), and FFM of 38.6 kg (IQR 32.3-47.1), respectively. Four participants (2%) were HIV positive. Cycloserine prescriptions were components of individualized, susceptibility-guided multidrug MDR-TB treatment regimens at the time of PK sampling. Co-administered MDR-TB treatments included linezolid (91% of participants), moxifloxacin (90%), clofazimine (79%), pyrazinamide (42%), ethambutol (34%), kanamycin (20%), para-aminosalicylic acid (25%), bedaquiline (28%), and ethionamide (22%). Most participants (>80%) took cycloserine doses with food at each PK visit. Table 1 shows the study participants’ baseline characteristics, which were not significantly different between participants with intensive and sparse PK sampling data. Cycloserine treatment duration and the schedule of the PK visits for each participant are shown in Fig. 1. During the first PK visit, most participants were prescribed cycloserine 250 mg BID (85% of participants) or 250/500 mg a.m./p.m. (9%). Prescriptions at the first PK visit for each participant are shown in Fig. S1 and indicate that intensive sampling represented lower-than-expected doses of cycloserine for heavier patients. MICs were determined in a total of 171 cultured isolates and exhibited a median value of 16 mg/L (range: 1–64 mg/L; see Fig. 3).

**TABLE 1:** Characteristics of the study population^*b*^

Variable (units)	*n* = 180
Age (years)	27 (21–35)
Females (*n*)	117 (65.0)
Weight (kg)	55.5 (46.0–65.9)
Height (m)	1.59 (1.53–1.69)
Fat-free mass (kg)	38.7 (32.3–47.1)
Serum creatinine (mg/L)	0.7 (0.6–0.9)
Creatinine clearance (mL/min)^*a*^	108 (91.7–133.0)
Current smoker (*n*)	8 (4.5)
HIV positive (*n*)	4 (2.2)

^*a*^ Cockcroft–Gault creatinine clearance estimates.

^*b*^ Data are presented as median (interquartile range) or number (percent). No significant differences were identified between the two sampling groups for categorical or continuous covariates evaluated with Fisher’s exact test and Wilcoxon’s signed-rank test, respectively.

### Pharmacokinetic model

The full study data comprised a total of 1,281 cycloserine observations, of which 312 were from intensive and 969 from sparse sampling. Imputation was employed for three BLD values (0.2%).

Cycloserine PK data were best described by a one-compartment disposition model with a first-order elimination and transit compartment absorption. Allometric scaling using FFM best described the effect of body size (∆OFV 20.9) on the disposition parameters since it proved a better fit than using total body weight (∆OFV 12.8). Inclusion of RF and FFM allowed the estimation of two elimination pathways (∆OFV 8.95, 1 df, *P* < 0.005) and demonstrated a better fit than Cockcroft–Gault creatinine clearance estimates without allometry (∆OFV 6.61, one df, *P* < 0.02). For a typical male with an FFM of 38.6 kg and a standard creatinine clearance of 122 mL/min, cycloserine CLr was estimated at 0.589 L/h, and CLnr was estimated at 0.901 L/h. We found no drug–drug interactions between cycloserine and other TB drugs, or any effect of total daily dose, HIV, administration with food, or smoking.

**Fig 2:**
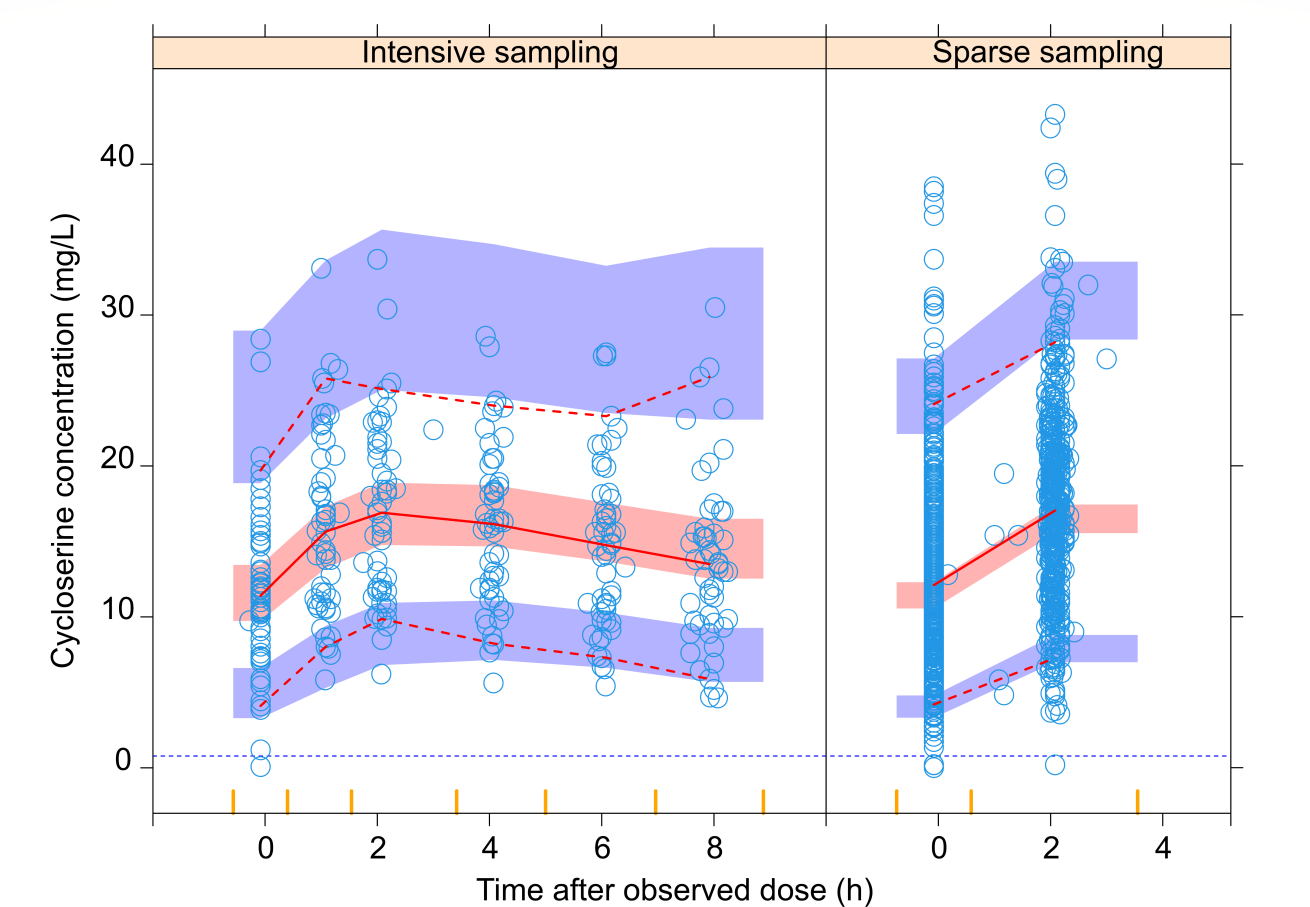
Visual predictive check from the final model. The solid and dashed lines represent the 5th, 50th, and 95th percentiles from the original observations (blue circles), while the shaded areas correspond to the 95% confidence intervals for the same percentiles based on model predictions for the intensively sampled data (left panel) and the sparsely sampled data (right panel). The horizontal dotted line represents the lower limit of quantification (0.782 mg/L). The bins for sampling points are shown as vertical yellow ticks on top of the plot area.

Between-subject variability in CL was included on both elimination routes as a single random effect, while between-occasion variability affected all absorption parameters (bioavailability, absorption rate constant, and mean transit time). The model fit significantly improved (∆OFV 83.8, 1 df, *P* < 0.001) after incorporating a factor to account for unobserved home doses, increasing their between-occasion variability for all absorption parameters by 2.26-fold. Table 2 shows the final PK parameters, and Fig. 2 demonstrates adequate model fit by visual predictive check.

**TABLE 2:** Final population pharmacokinetic parameters^*g*^^,*h*^

Parameter	Typical value (95% CI^*e*^)	Variability^*f*^ (95% CI^*e*^)
Renal clearance (L/h)^*a*^^,*b*^	0.589 (0.449–0.745)	BSV: 34.1 (30.2–38.7)
Non-renal clearance (L/h)^*a*^	0.901 (0.767–1.04)	
Central volume (L)^*a*^	37.0 (35.1–38.9)	NA
Absorption rate constant (1/h)	2.15 (1.51–3.55)	BOV: 94.6 (79.4–121)
Mean transit time (h)	0.610 (0.362–0.833)	BOV: 83.5 (67.3–111)
Number of transit compartments (NN)^*c*^	4 fixed	NA
Bioavailability	1 fixed	BOV: 20.7 (18.9–22.7)
Variability factor for unobserved doses (fold change)^*d*^	2.26 (1.97–2.54)	NA
Proportional error (%)	4.58 (3.23–5.66)	NA
Additive error (mg/L)	0.856 (0.716–1.03)	NA

^*a*^ All disposition parameters were allometrically scaled to an individual with a fat-free mass of 38.6 kg.

^*b*^ Renal clearance was estimated for a typical male with standard creatinine clearance of 122 mL/min.

^*c*^ NN was fixed to 4 to improve the stability of parameter estimates (a sensitivity analysis confirmed that this did not significantly affect the final model fit).

^*d*^ The scaling factor applies to the BOV of absorption rate constant, mean transit time, and bioavailability for self-reported doses taken at home on days prior to blood sampling.

^*e*^ SIR was used to obtain the 95% CI.

^*f*^ BSV and BOV were assumed to be log-normally distributed and reported as percent coefficient of variation (% CV) calculated by $\%\;CV=\sqrt{\omega^{2}}\times 100.$

^*g*^ BOV, between-occasion variability; BSV, between-subject variability; CI, confidence interval; SIR, sampling importance resampling.

^*h*^ N/A indicates not applicable.

### Probability of target attainment

**Fig 3:**
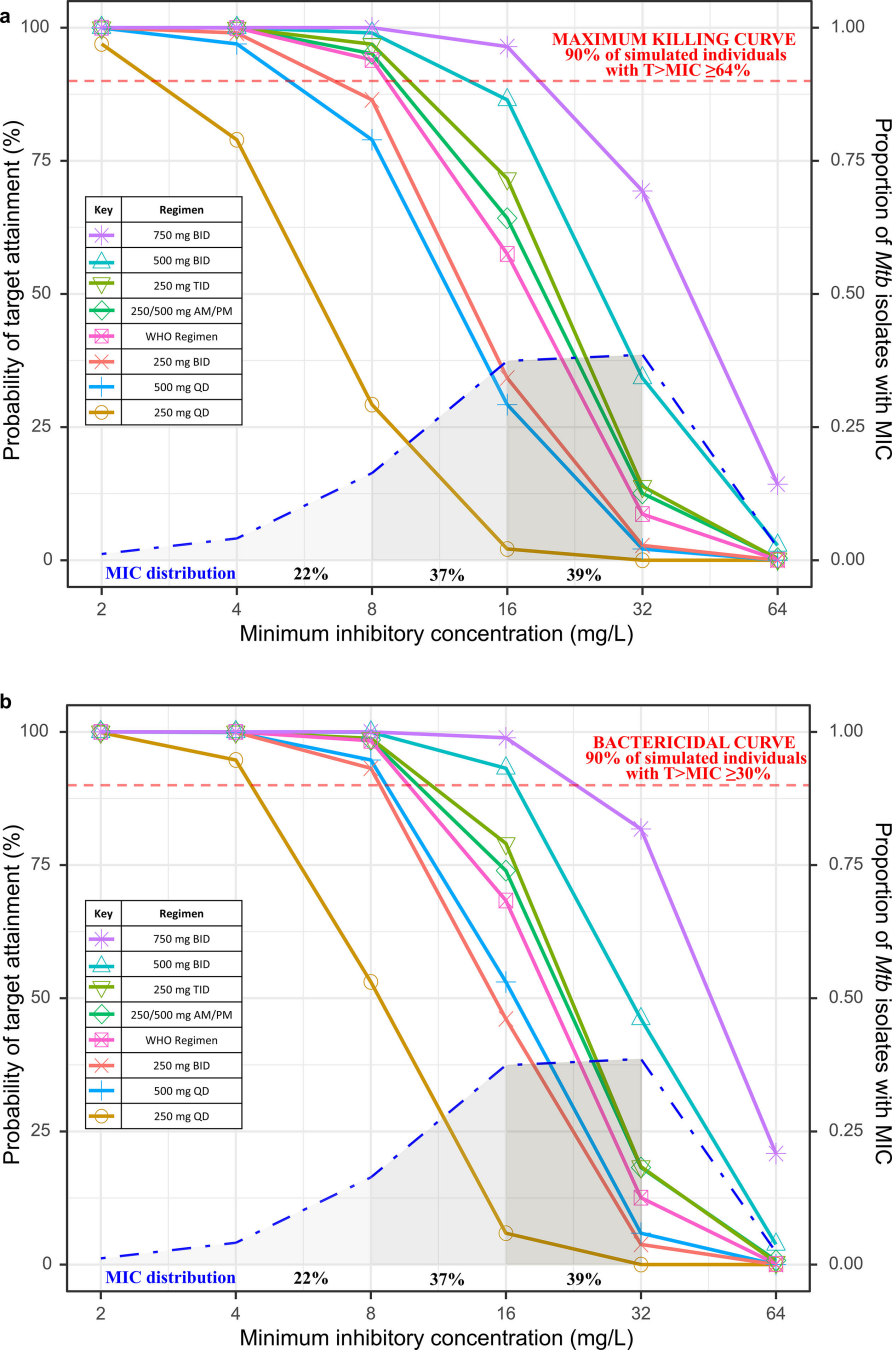
Probability of target attainment by dose and minimum inhibitory concentration. Solid lines indicate the probability of target attainment on the primary *y*-axis (on left) based on the percentage of time during the dose interval in which the concentration exceeds the minimum inhibitory concentration (%*T*_>MIC_) on the *x*-axis. The horizontal dashed red lines indicate the 90% attainment. The top panel shows (**a**) %*T*_>MIC_ of ≥ 64% (the preferred literature-derived target indicating 80% maximum kill effect) and the bottom panel (**b**) or %*T*_>MIC_ of ≥ 30% (bactericidal effect). Solid lines indicate simulated cycloserine doses of 250 mg QD, BID, or TID; 500 mg QD or BID; 250/500 mg a.m./p.m.; 750 mg BID; and WHO weight-based regimen (250 mg BID for body weights ≤45 kg and 250/500 mg a.m./p.m. for weights >45 kg, as indicated by color and shape in the legend. The dot-dashed curve and shaded area underneath indicate the distribution of cycloserine MICs for *Mycobacterium tuberculosis* isolates cultured from 171 study participants (the proportion of MIC in tested samples can be read on the right *y*-axis).

The PTA for bactericidal activity (*T*_>MIC_ ≥30%) and 80% maximum kill (*T*_>MIC_ ≥64%) for the simulated regimens using the median Cockcroft–Gault creatinine clearance estimate of 109 mL/min is shown in Fig. 3. For isolates with lower MICs of ≤8 mg/L (representing 22% of isolates tested), >90% of simulated individuals were predicted to attain *T*_>MIC_ of ≥30% with any of the regimens evaluated except for 250 mg daily, and this target could be achieved with regimens as low as 250 mg BID or 500 mg QD. For the median cycloserine MIC value of 16 mg/L (37% isolates tested), only doses of 500 and 750 mg BID had high probabilities of target achievement (93% and 99%, respectively). With regimens of 250 mg TID and 250/500 mg a.m./p.m., around 75% of the simulated individuals reached this target, while the WHO-recommended weight-banded approach achieved this target for 68% of simulated individuals. The PTA with lower doses of 500 mg QD and 250 mg BID was lower at ~50% of the virtual population, while a dose of 250 mg QD only achieved *T*_>MIC_ of ≥30% for 6% of simulated subjects.

Using the preferred target of *T*_>MIC_ ≥64%, achieving 80% of maximum kill, Fig. 3 demonstrates similar trends, but only cycloserine 750 mg BID surpassed 90% PTA (96%) for an MIC of 16 mg/L, with 500 mg BID nearly approximating this goal (86% PTA) and lower doses, such as the WHO-recommended weight-banded regimen, 250 mg TID, and 250/500 mg a.m./p.m. achieving lower PTA (57%, 71%, and 64%, respectively).

Figure S2 shows the PTA stratified by Cockcroft–Gault creatinine clearance estimates for the bactericidal and maximum kill targets.

### Observed and simulated exposures and toxicity

The cycloserine maximum concentrations observed in our intensive data were reached at a median time of 2 h (IQR 1.2–2.2 h) post-dose and ranged from 6.21 to 26.8 mg/L with a median value of 16.8 mg/L for regimens of 250 mg BID and from 27.3 to 33.7 mg/L with a median value of 30.5 mg/L for regimens of 250/500 mg a.m./p.m. A dose-proportional increment in cycloserine exposures was observed with a higher dose level. The observed median cycloserine AUC_0–24_ values in individuals receiving 250 mg BID and 250/500 mg a.m./p.m. were 347.4 mg·h/L (IQR 261.1–425.7) and 415.7 mg·h/L (IQR 224.1–553.9), respectively.

A total of 581 AUC values were evaluated, of which 15 values (2.6%) from seven participants (3.9%) exceeded a literature-derived AUC toxicity threshold of 700 mg·h/L (11). Cycloserine toxicity was not more frequent among those above the threshold (*N* = 15 evaluations) than those below (*N* = 566) at the time of drug-level measurement, including depression (6.7% vs 4.9%, *P* = 1.00), neuropathy (46.7% vs 29.3%, *P* = 0.25), and psychosis (6.7% vs 3.5%, *P* = 1.00). Similarly, participants with AUC >700 mg·h/L did not report depression at any point during their treatment more frequently than those whose AUC remained below 700 mg·h/L (42.9% vs 37.6%, *P* = 1.00), nor did they more frequently report neuropathy (85.7% vs 66.2%, *P* = 0.52) or psychosis (28.6% vs 30.1%, *P* = 1.00) symptoms at any time during their 3-year follow-up. When we evaluated the estimated log2(AUC) as a continuous variable against contemporaneous report of toxicity, we found no significant association between high AUC and either neuropathy or psychosis. We did, however, find a significant association indicating increased odds of higher-grade depression (PHQ-9 >10 or >14, details in supplement) as AUC increased. When looking more closely at these trends, we found that the minority of cohort participants with higher AUCs reported toxicity, with a greater proportion of those with high AUCs denying such symptoms. Consistent with this, when using a receiver operating characteristic (ROC) analysis to discriminate those with and without depression, the best AUC threshold identified was above the median values for both those with and without depression and achieved poor sensitivity to identify those with depression (sensitivity 39%–50%, depending on PHQ9 threshold applied). A broader explanation of this analysis with figures and tables is presented in the supplemental material (Fig. S3).

**Fig 4:**
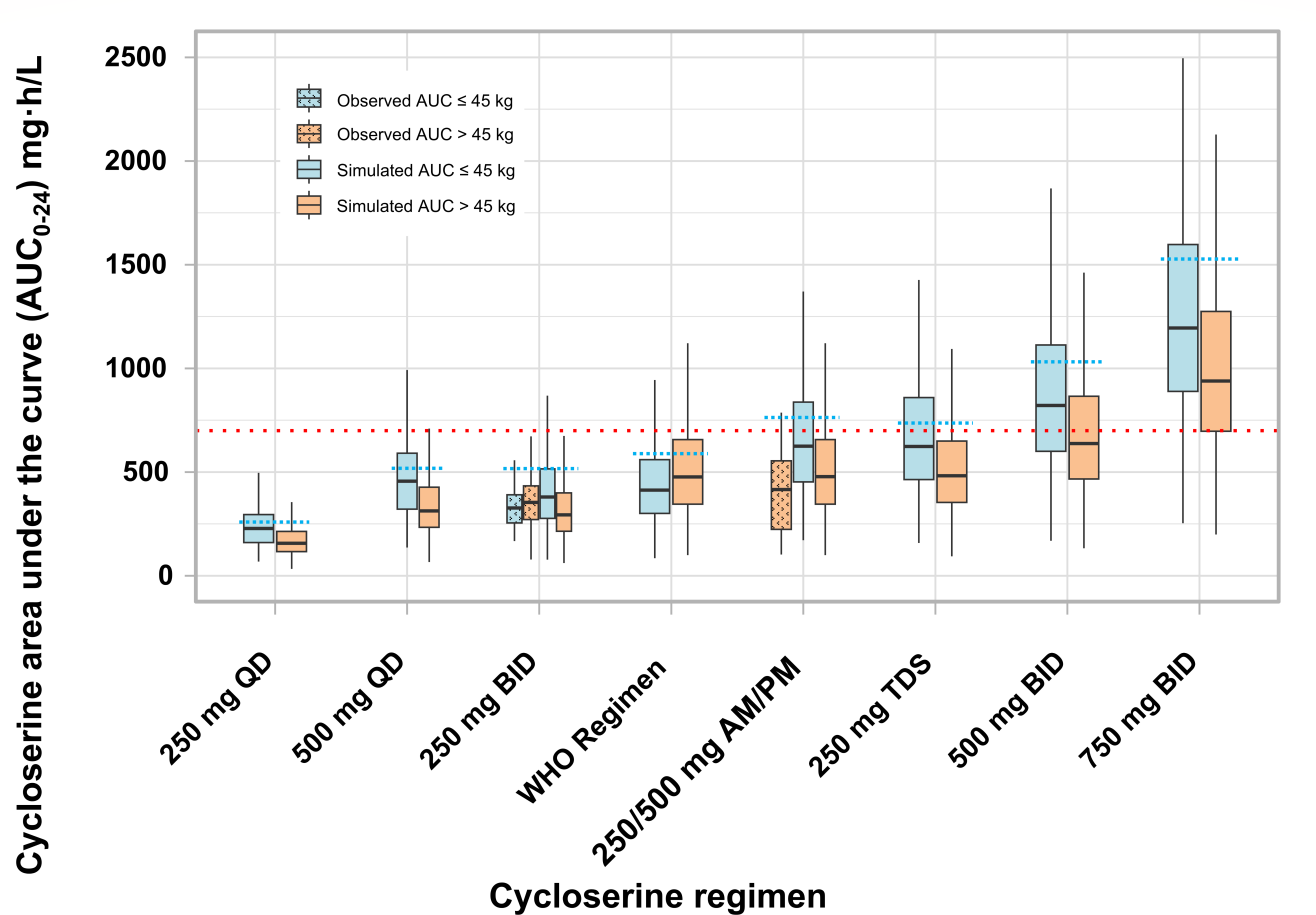
Observed and simulated cycloserine area under the curve (AUC_0−24_) at different dose regimens. Boxes represent the median and interquartile range of the cycloserine area under the curve (AUC_0–24_), while the whiskers show the range of values. Observed exposures are shown as boxes with divot pattern fill, while the simulated are shown as solid boxes. The horizontal red dotted line indicates the literature-derived toxicity threshold AUC_0–24_ of 700 mg·h/L associated with toxicity (11). The horizontal dotted blue lines on top of each group of boxes represent the reported exposure for the specific dose regimen (12, 14).

Figure 4 compares both the observed exposures in our study cohort and model-based simulations with the AUC toxicity threshold reported by Court et al. (11) and previously reported exposures for each dosing regimen by Alghamdi et al. (12) and Chirehwa et al. (14). Overall, the observed exposures in our study, as well as the simulated values with our final model, were lower than previous findings at each dose level (Table 3).

**TABLE 3:** Median observed and simulated cycloserine area under the curve (AUC_0–24_) per dose regimen^*d*^*^,^*^*e*^^,^^*f*^

Cycloserine dose	Observed AUC_0–24_	Simulated AUC_0–24_	Reference value
250 mg QD	NA	169.8 (123.1–239.1)	259.5 (97.9)^*b*^
500 mg QD	401.5^*a*^	339.7 (246.2–478.1)	519.0 (195.7)^*b*^
250 mg BID	347.4 (261.1-425.7)	308.6 (226.2–432.3)	516.7 (188.3)^*b*^
250/500 mg a.m./p.m.	415.7 (224.1-553.9)	506.9 (363.6–700.4)	763.8 (271.0)^*b*^
250 mg TID	NA	513.4 (373.5–694.1)	737.7 (239.1)^*b*^
500 mg BID	NA	670.9 (491.7–939.8)	1,033.4 (376.7)^*c*^
750 mg BID	NA	1,006 (737.5–1410)	1,527.5 (537.3)
WHO regimen	NA	462.0 (331.8–629.4)	587.0 (175–1451)

^*a*^ AUC during the 500 mg QD regimen was observed only in one participant.

^*b*^ Reference values are shown as originally reported, mean and standard deviation from Alghamdi et al. (12).

^*c*^ Reference values are shown as originally reported, median and range from Chirehwa et al (14).

^*d*^ QD, once daily, BID, twice daily, TID, thrice a day. AUC_0–24_, cycloserine area under the concentration curve.

^*e*^ Observed and simulated AUC_0–24_ are shown as median and interquartile range.

^*f*^ N/A indicates not applicable.

## DISCUSSION

We developed a model describing the population PK of cycloserine in a cohort of Indian adults and adolescents with MDR-TB. The model identified the effect of body size using FFM and that of renal function by separating CLr and CLnr. Overall, we observed cycloserine concentrations much lower than previously reported with the same dosing regimens. Consistently, Monte Carlo simulations predicted that dosing with 500 or 750 mg BID would be necessary for >85% of individuals to achieve the drug exposure required for 80% maximum kill at the median MIC value in this cohort of 16 mg/L. However, for more susceptible isolates with MIC of 8 mg/L (22% of samples), dosing options such as 250 mg TID, split doses of 250/500 mg a.m./p.m., or WHO weight-banded regimens would also meet the %*T*_>MIC_ targets.

Cycloserine exposure observed in this study was markedly lower than reported in previous studies. Specifically, the median maximum concentrations in our data were 16.8 mg/L for the 250 mg BID regimen and 30.5 mg/L for the 250/500 mg a.m./p.m. regimen, compared to reported values of 26.4 and 42.2 mg/L, respectively, for the same dosing regimens (12). Likewise, the observed AUC_0–24_ in participants receiving 250 mg BID was lower compared to one reported in a multicountry (Georgia, Bangladesh, and USA) population (342.9 vs 516.7 mg·h/L), also for those receiving 250/500 mg a.m./p.m. (446.2 vs 763.8 mg·h/L). The same trend was found for all the simulated dose regimens with our final model (12).

Our cycloserine PK model had one-compartment disposition, first-order elimination, and absorption with transit compartments, which is concordant with previous studies (12, 14, 31). Our model supports the estimation of both non-renal and renal elimination pathways, with CLr representing 40% of total CL. The contribution of the renal elimination pathway observed in this study was lower than the 60%–70% previously suggested by Welch et al. (9) and the 55% reported by Chirehwa et al. among South African participants receiving terizidone (14). Interestingly, the total CL and volume of distribution observed in this Indian cohort were higher than those previously reported in populations with similar FFM and body weight. Chirehwa et al. reported a total CL of 0.776 L/h and a volume of distribution of 23.2 L in a South African population, both allometrically scaled with FFM (14). On the other hand, Alghamdi et al. described the effect of size based on weight, with CL estimated at 1.03 L/h and volume of distribution at 24.9 L in a multinational population study (12). The higher total CL (and higher contribution of the CLnr pathway) is consistent with the lower exposures in our study population. It has been suggested that CLnr increases by 41% among smokers (14), possibly related to induction of cytochrome P450 enzymes (32), but this was uncommon in our study (only 4.5% in this cohort reported tobacco use) and was not found to significantly impact CLnr. On the other hand, since we observed larger values of both CL/F and V/F, the overall difference may be explained by lower bioavailability. This may reflect differences in cycloserine formulations, but in our study, all participants used the same Lupin formulation, so we could not investigate this.

Cycloserine is sometimes dosed as terizidone, which increases time to maximum concentration (*T*_max_) to 5 h (14), longer than the 2 h *T*_max_ achieved with cycloserine (12). *T*_max_ is also increased when cycloserine is administered with food (33), but *T*_max_ in our study was consistent with the 2 h reported in the literature, despite >80% of study participants taking cycloserine with food. Another potential source of the differences in observed exposure compared to reported values could be assay variability. However, to ensure the quality of our cycloserine observations, bilateral sample exchange with an international collaborator confirmed the accuracy of the low levels observed in our study, ruling out lab error as a cause of the discrepancy (data not shown).

Cycloserine efficacy depends on time above MIC (10). While most TB drugs are tested for susceptibility at a single critical concentration, no critical concentration for cycloserine testing is currently endorsed by the WHO, so susceptibility testing is an uncommon practice worldwide (34, 35). Proposed cycloserine critical concentrations include values of 16 mg/L on MGIT 960 systems (35) and from 25 to 40 mg/L on Löwenstein–Jensen (34, 36). Interpretation of susceptibility tests is further limited by cycloserine degradation over time, limiting reproducibility and correlation of susceptibility patterns with clinical results (36). Importantly, no genotypic markers of cycloserine resistance have yet been validated, so treatments must be either empiric or rely on imperfect phenotyping (37). Among isolates in our population, 76.5% had MIC values ≥ 16 mg/L, which is similar to distributions reported elsewhere (14, 38). As a result, empiric regimens should target the most common “susceptible” phenotype, with an MIC of 16 mg/L unless isolates are confirmed to be more susceptible. Using the model we developed, the most reliable regimens to achieve maximum efficacy (*T*_>MIC_ ≥64%) would be 500 mg BID or greater, though approximately 75% of simulated individuals could achieve the lower bactericidal targets with doses of either 250 mg TID or 250/500 mg a.m./p.m. While lower doses are often used clinically out of concern for toxicity, this study suggests that they may provide suboptimal dosing for Indian patients, with no clear association between exposure and increased toxicity in this cohort.

We previously reported lower-than-expected incidence of neuropsychiatric toxicity among the participants taking cycloserine at doses of up to 250/500 mg a.m./p.m. in this study, which aligns with the finding of lower-than-expected drug concentrations observed in the present analysis and is supported by both the small number of participants with elevated AUC in the current analysis and the infrequent toxicity among those with higher AUC (5). In our cohort, a previously reported threshold (AUC >700 mg·h/L) was not associated with the development of any of the cycloserine-associated toxicities we evaluated nor was AUC as a continuous variable associated with concurrent neuropathy or psychosis. While AUC as a continuous variable was statistically significantly higher among those with more severe depression than those without, cycloserine concentrations between those with and without toxicity overlap. This means that it was not possible to identify a discriminating threshold by ROC analysis that achieved reasonable sensitivity, suggesting that this was not a meaningful finding in our data set. Consistent with our previous findings (5) and despite concerns, PHQ-9 scores were not higher among those taking cycloserine compared to those who did not, nor were PHQ-9 scores higher among those taking higher doses of cycloserine compared to those taking lower doses. This may reflect uncertainty in the relationship between plasma drug concentration and clinical toxicity, but the combination of lower-than-expected drug levels and lower rates of depression and neuropathy in this cohort suggests that while clinicians must remain vigilant for clinical toxicity during therapy, higher doses may have been better tolerated than expected in this cohort.

Our analysis has some limitations. Results of this single-site study may not be generalizable to other populations, including non-Indian populations and those with higher rates of comorbid HIV, diabetes, or malnutrition. Likewise, the availability of only a single formulation of cycloserine provided to study participants may have contributed to the low observed drug levels and limited our ability to make comparisons. In addition, only a subset of study participants underwent intensive sampling, and the self-reported dosing history increased the observed variability of pre-dose trough concentrations. Follow-up studies including either in-person or smartphone-based direct observation of therapy may improve on the interpretation of these values (39). The PK targets we employ in this study were derived from hollow-fiber PK models (10), which still require validation in humans to correlate bactericidal activity with clinical outcomes. While this analysis suggests an opportunity to further optimize doses without increasing toxicity, in light of the relatively low rate of all-cause mortality and relapse in this MDR-TB cohort (8 out of 180 participants, 4.4%, and 1 out of 180, 0.6%), failure to meet the PK targets described does not mean that treatment was ultimately unsuccessful. Making the extrapolation of these targets to humans even more challenging is the scarcity of information on the plasma protein binding of cycloserine, which was assumed to be 100% free. While we performed MIC testing using quality-controlled plates, cycloserine can degrade over time, so it is possible that our MIC interpretation may be artificially inflated (36). If cycloserine is to remain an important component of MDR-TB treatment, improved susceptibility testing methods such as a fixed critical concentration or molecular testing could improve the evaluation of its efficacy. Another limitation is the lack of genetic and urine data that could have been useful to corroborate the possibility of higher cycloserine metabolism or the lower renal elimination component found in this study.

In conclusion, we described the population PK of cycloserine among Indian adults and adolescents treated for MDR-TB, quantifying the effect of body size and renal function on clearance. We observed lower concentrations than previously reported, possibly due to larger values of clearance or lower bioavailability in this cohort. The reasons for this are unclear, but future studies are needed to confirm and investigate this, maybe focusing on the impact of pharmacogenetic factors. It should further be investigated if individualized dosing based on population PK models and bug susceptibility may improve treatment outcomes for MDR-TB.

## AFTER EPUB

*[This article was published on 2 September 2025 with an error in the author contributions. The author contribution was corrected in the current version, posted on 1 October 2025.]*

## SUPPLEMENTAL MATERIAL

Supplemental material aac.00101-25-s0001.docx Fig. S1 to S3; Tables S1 and S2.

ASM does not own the copyrights to Supplemental Material that may be linked to, or accessed through, an article. The authors have granted ASM a non-exclusive, world-wide license to publish the Supplemental Material files. Please contact the corresponding author directly for reuse.
