# Supplementary material for: Evaluation of cycloserine dose regimens in an Indian cohort with multidrug-resistant tuberculosis: a population pharmacokinetic analysis
Source: Antimicrob Agents Chemother. 2025 Sep 2;69(10):e00101-25. doi: 10.1128/aac.00101-25 (PMC12486832; doi:10.1128/aac.00101-25)
Supplement: Supplemental material — Fig. S1 to S3; Tables S1 and S2. [file aac.00101-25-s0001.docx]

Evaluation of cycloserine dose regimens in an Indian cohort with multidrug-resistant tuberculosis: a population pharmacokinetic analysis.

Juan Eduardo Resendiz-Galvan* (1), Prerna R. Arora* (2), Rohan V. Lokhande (2), Zarir F. Udwadia (2), Camilla Rodrigues (2), Amita Gupta (3-5), Jeffrey A. Tornheim (3-5)^†^, Paolo Denti (1)^†^, and Tester F. Ashavaid (2)^†^ for The MDR-TB MUKT and Indo-South Africa Study Teams


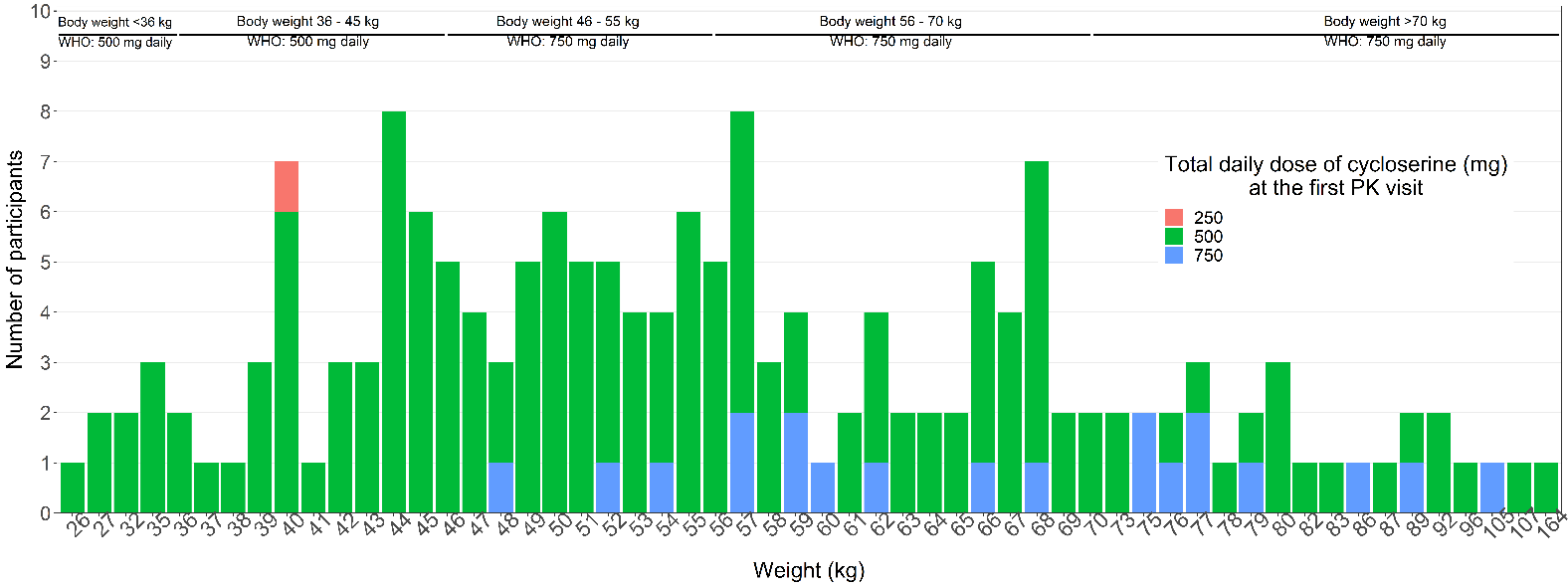
Figure S1. Total daily dose of cycloserine prescribed for each participant during their first pharmacokinetic visit.

The upper legends represent the weight bands, and the corresponding doses recommended by WHO. The total daily doses are represented by different colours. The bars at each weight indicate the number of participants (increments by 1 on the y-axis) receiving a specific dose.

Figure S2. Probability of target attainment by dose, minimum inhibitory concentration, and Cockcroft-Gault creatinine clearance estimates.


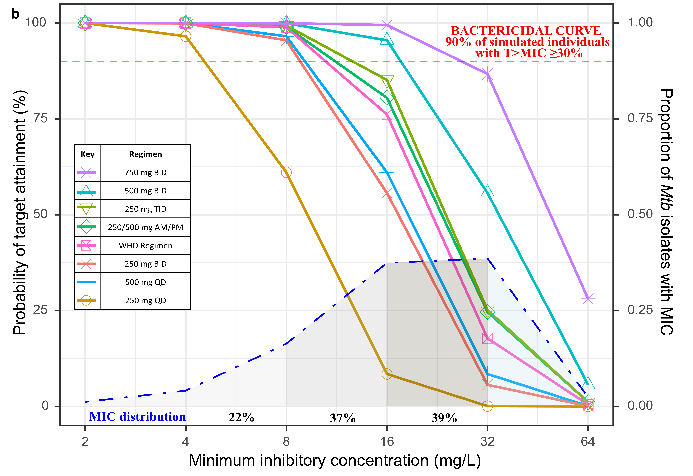

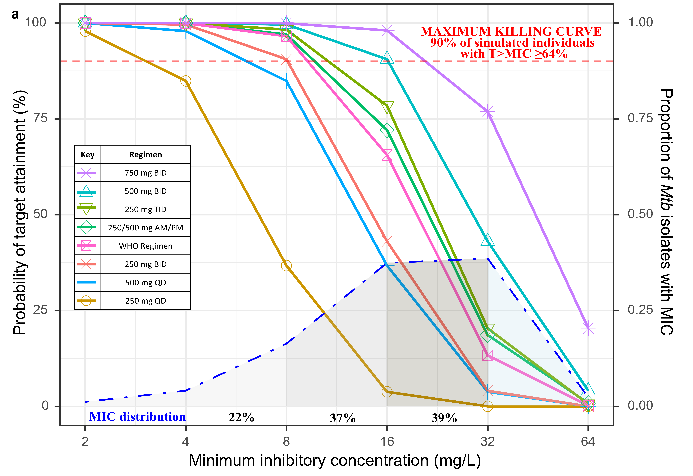
Creatinine clearance = 91.1 mL/min (Q1)


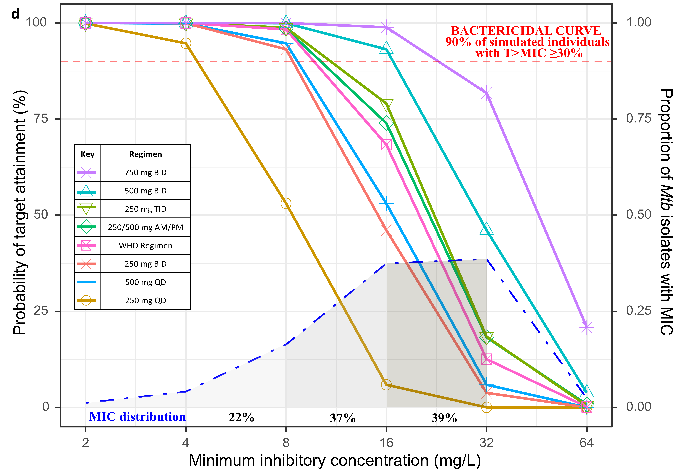

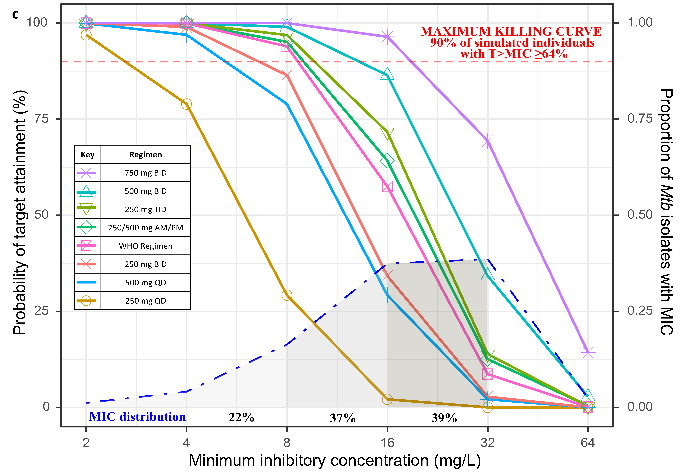

Creatinine clearance = 109 mL/min (Median)


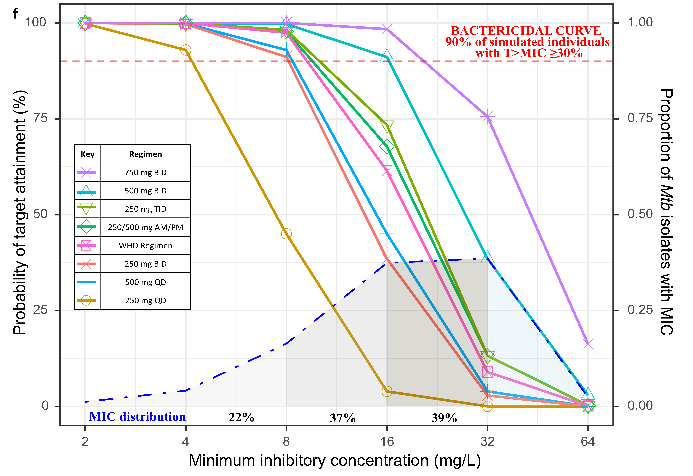

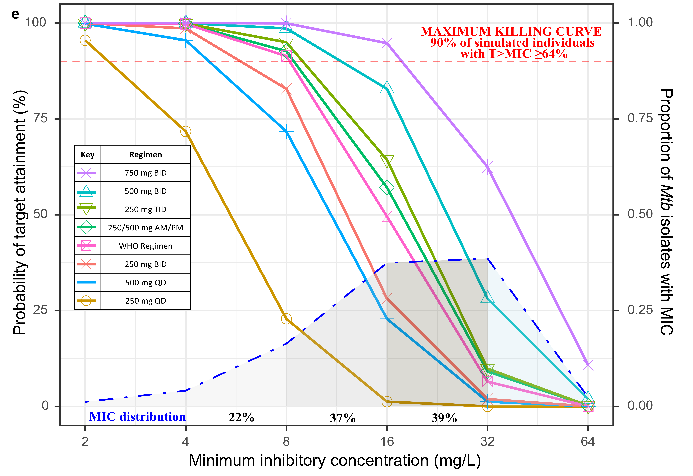
Creatinine clearance = 133 mL/min (Q3) (FiguresS2e and S2f)

Solid lines indicate the probability of target attainment (PTA) on the primary y-axis (on left) based on the percentage of time during dose interval which the concentration exceeds the minimum inhibitory concentration (%T_>MIC_) on the x-axis. The PTA is stratified into different levels based on Cockcroft-Gault creatinine clearance estimates (median [S2c-d] and interquartile range [Q1 (S2a-b), Q3 (S2e-f),]) from the full population. The horizontal dashed red lines indicate the 90% of attainment when %T>MIC ≥64% (the preferred literature-derived target indicating 80% maximum kill effect, left panel [S2a, c, e]) or ≥30% (bactericidal effect, right panel [S2b, d, f). Solid lines indicate simulated cycloserine doses of 250 mg QD, BID, or TID; 500 mg QD or BID; 250/500 mg AM/PM; 750 mg BID; and the proposed regimen by WHO consisting of 500 mg for body weights ≤45 kg and 750 mg for body weights ≥46 kg, as indicated by colour and shape in the legend. The dot-dashed curve indicates the distribution of cycloserine MICs for *Mycobacterium tuberculosis* isolates cultured from 171 study participants. The shaded area under the dot-dashed line indicates the proportion of tested samples with the corresponding MIC.

**Evaluation of cycloserine toxicity**

To evaluate the relationship between cycloserine exposure and toxicity we estimated the AUC for all participants in the cohort at the time of their clinical evaluations and dichotomized the values as greater than or less than the literature-derived AUC toxicity threshold of 700 mg·h/L (Court R. et al. Int J Infect Dis. 2021). Then, we dichotomized each study participant as someone who did or did not ever have an elevated AUC value and performed univariate logistic regression (glm function, R) of elevated AUC values on the odds of cycloserine associated toxicity (outcomes, neuropathy, treatment neuropathy, depression measured by PH1Q9 scores, or psychosis). We did not find an association between elevated AUC values and the occurrence of any of the toxicities mentioned, as shown in the Table S1 below.

| Variable | Odds Ratio | p-value |
| --- | --- | --- |
| Neuropathy | 3.00 | 0.314 |
| Neuropathy Treatment | 2.69 | 0.205 |
| Any Depression | 1.29 | 0.742 |
| Any Severe Depression | 0.00 | 0.992 |
| Any Psychosis | 0.96 | 0.963 |
| Any Severe Psychosis | 0.00 | 0.995 |

Table S1. Association between elevated AUC and toxicity. Odd ratios of toxicity in participants with cycloserine AUC above the threshold of 700 mg·h/L.

In a separate analysis, the estimated AUCs were used raw and Log2 normalized continuous variables and performed univariate logistic regression (glm function, R) against simultaneous report of the toxicities at the time that the AUCs were elevated. This analysis found no significant between cycloserine AUC and either neuropathy or psychosis. However, there was a significant association between those with higher AUC and increased odds of higher severities of depression measured by PHQ9 >10 or PHQ9 >14. The results of this analysis are contained in Table S2, below.

| Variable | Odds Ratio | p-value |
| --- | --- | --- |
| Neuropathy | 1.17 | 0.33 |
| Depression (PHQ9 >10) | 3.27 | <0.001 |
| Depression (PHQ9 >14) | 4.34 | 0.001 |
| Any Psychosis | 1.62 | 0.24 |
| Any Severe Psychosis | 5.95 | 0.361 |

Table S2. Association between estimated cycloserine Log2 AUC as a continuous variable and toxicity. Odds ratios of toxicity per unit increase in Log2 AUC (every doubling of AUC values).

In a closer look to these trends (Figure S3), we found that the significant association was importantly driven by smaller numbers of high AUC outliers. However, there were more individuals with the same high AUC values who did not have that toxicity as shown in the figure below. This means that, while there is a significant association between higher AUCs and higher PHQ9 scores, the sensitivity of higher AUC to identify either current or future depression was inadequate to discriminate these groups by ROC analysis.


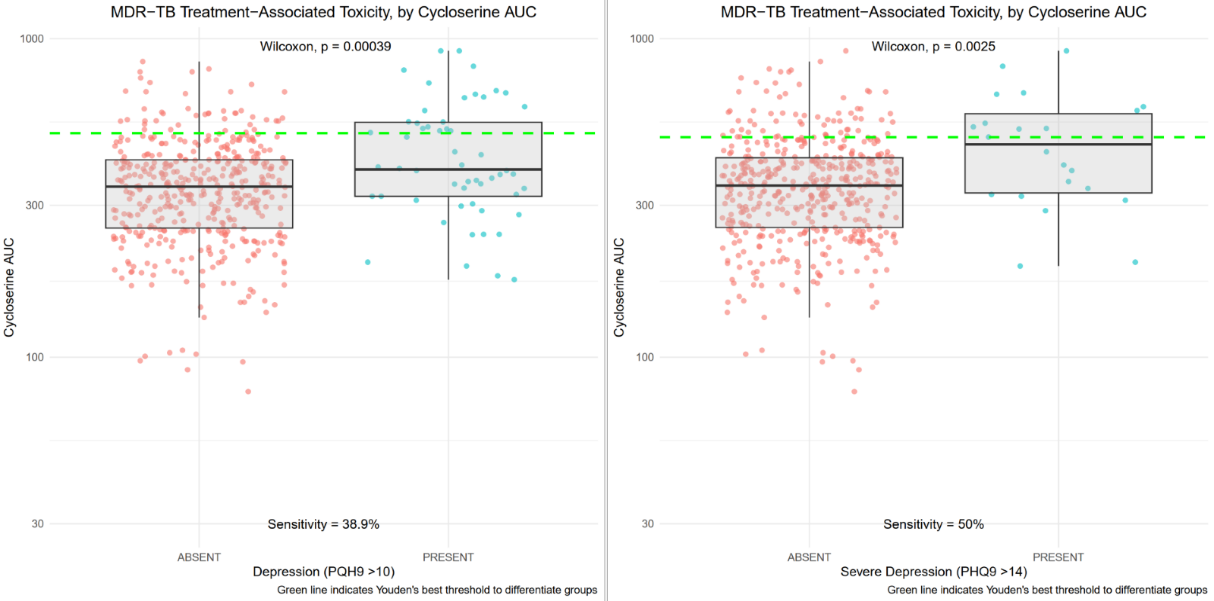
Using the pROC package in R, the Youden’s best threshold to use AUC as discriminator between those individuals with or without depression was above the median values for both groups and only reached a sensitivity to detect depression of 39-50%, depending on PHQ9 threshold applied.

Figure S3. Depression (moderate and severe on the left and right panel, respectively) vs. concomitant cycloserine Log2 AUC. The horizontal green dotted line represents the Youdens’s best threshold to discriminate the two groups.
